# Human parasitism by the exotic tick *Dermacentor variabilis* (Parasitiformes: Ixodida) in Brazil: report of an imported case

**DOI:** 10.1590/S1984-29612021093

**Published:** 2022-01-05

**Authors:** Thiago Fernandes Martins, Adriano Pinter

**Affiliations:** 1 Departamento de Laboratórios Especializados, Superintendência de Controle de Endemias, Secretaria de Estado da Saúde de São Paulo, São Paulo, SP, Brasil; 2 Departamento de Medicina Veterinária Preventiva e Saúde Animal, Faculdade de Medicina Veterinária e Zootecnia, Universidade de São Paulo, São Paulo, SP, Brasil

**Keywords:** Acari, Ixodidae, public health, Brazilian traveler, United States of America, Acari, Ixodidae, saúde pública, viajante brasileiro, Estados Unidos da América

## Abstract

In June 2012, a tick was found parasitizing a man in the city of São Paulo, who had recently returned from a visit to Pennsylvania, in the northeast of the United States. The tick was removed and sent to the São Paulo State Department of Health, where it was identified as a male of the species *Dermacentor variabilis* (Say, 1821), according to the literature and taxonomic keys. The tick was subjected to a PCR test to search for rickettsiae, but the result was negative. The fact that a human entered Brazilian territory unaware that he was parasitized by a hard tick not belonging to the national tick fauna is significant because of the possibility that an exotic species could be introduced and take hold in this country. Another major risk to public health is that this arthropod could be infected with the bacterium *Rickettsia rickettsii*, as this ectoparasite is the main vector of Spotted Fever on the East Coast of North America.

## Resumo

Em junho de 2012, foi enviado ao serviço da Secretaria de Estado da Saúde de São Paulo um carrapato que foi encontrado em parasitismo sobre um homem adulto na cidade de São Paulo, que havia chegado recentemente de uma viagem de turismo aos Estados Unidos, onde visitou o estado da Pensilvânia, situado na região Nordeste Americana. O carrapato foi identificado como um macho da espécie *Dermacentor variabilis*, (Say, 1821), de acordo com a literatura e chaves taxonômicas, sendo submetido ao teste da PCR para pesquisa de riquétsias, porém o resultado foi negativo. O fato de um ser humano ter cruzado a fronteira do Brasil, parasitado, sem o seu prévio conhecimento, por um carrapato duro, não pertencente à ixodofauna nacional, é de grande importância pela chance de introdução e estabelecimento no território brasileiro de uma espécie exótica. Outro grande risco para a saúde pública é que esse artrópode poderia estar infectado com a bactéria *Rickettsia rickettsii*, pois esse ectoparasito é o principal vetor da Febre Maculosa na costa Leste Norte Americana.

The hard tick *Dermacentor variabilis* (Say, 1821) occurs throughout Canada, the United States of America and Mexico, parasitizing a large diversity of wild and domestic mammals during its adult stage, and mainly rodents during its immature stages (Guglielmone et al., 2014; Guglielmone & Robbins, 2018). In addition to being of veterinary importance, it is of medical importance because it accidentally parasitizes humans and is a well-known vector of the bacterium *Rickettsia rickettsii*, the etiological agent of Rocky Mountain Spotted Fever (Furman & Loomis, 1984; Guglielmone & Robbins, 2018). Few studies to evaluate the vector competence of *D. variabilis* to transmite of *Borrelia burgdorferi* sensu lato have been carried out so far, whereby it has not been experimentally confirmed this tick species as a spirochete vectors (Eisen, 2020).

In Brazil, reports of local native ticks parasitizing human beings are common, particularly the species belonging to the genus *Amblyomma* (Guglielmone & Robbins, 2018). On the other hand, reports of imported exotic ticks parasitizing humans in Brazilian territory are rare and are usually associated with travelers (Serra-Freire et al., 2015; Faccini-Martínez et al., 2021).

The objectives of this study were two-fold. One, to offer the first report in Brazilian territory, to the best of our knowledge, of parasitism by the tick *D. variabilis* on a Brazilian tourist returning to his home country after a visit to the United States of America (USA). And secondly, to determine the presence or absence of rickettsiae in this tick specimen.

On July 5, 2012, the office of Brazil’s National Health Surveillance Agency (ANVISA) at São Paulo International Airport forwarded a tick to the Superintendence of Endemic Disease Control (SUCEN) of the São Paulo State Department of Health (SES/SP).

The tick was found parasitizing an adult man who was a resident of the city of São Paulo, Brazil, upon his arrival at the aforementioned airport after a 9-hour overnight flight from the USA, where he was traveling in the state of Pennsylvania, located in the northeastern region of the country. The man noticed the tick attached to his skin after disembarking from the aircraft, whereupon he went to the office of ANVISA airport to report the finding. The tick, the only one found on the newly arrived passenger, was removed and stored in pure ethanol. No further information about was obtained about which cities he had visited or if he had been traveling in rural or forest areas.

The tick was examined under a LEICA M205 C stereomicroscope at the Laboratory of Medical Entomology of SUCEN, and was identified based on specific taxonomic keys and the species’ morphological characteristics published in the literature (Cooley, 1938; Arthur, 1960; Furman & Loomis,1984). In addition, the specimen sent by ANVISA to SUCEN was analyzed by PCR, targeting the *glt*A gene, primers CS-5 and CS-6, to determine the presence or absence of *Rickettsia* of the Spotted Fever group, as described by Labruna et al. (2004).

An acarological analysis indicated the specimen was a male of the species *D. variabilis* and the PCR test showed it was negative for Spotted Fever Group *Rickettsia*. The taxonomic and rickettsial findings were sent to the man who reported the discovery of the tick, and although the especimen was negative for rickettsial infection, he was asked to check for spotted fever symptoms for the next 15 days. In addition, all the passenger that had sat next to him or in the rows in front and behind him on the flight were contacted and asked to check for signs of spotted fever. However, these passengers reported no symptoms.

According to Estrada-Peña & Jongejan (1999), this tick is often found parasitizing humans. However, Guglielmone & Robbins (2018) state that the immature stages of this tick species are rarely found on humans, although adults ticks are commonly reported. Therefore, the human parasitism by an adult tick found in this study is in agreement with data in the literature. The occurrence of human parasitism is also corroborated by other works in the literature that reported travelers parasitized by *D. variabilis* in Australia, Panama and New Zealand (Halliday & Sutherst, 1990; Bermúdez et al., 2010, 2019; Heath & Hardwick, 2011).

To date, 14 exotic tick species have been reported in Brazil but have probably not become established, including species of several genera: *Amblyomma argentinae* Neumann, 1905, *Amblyomma crassum* Robinson, 1926, *Amblyomma darwinii* Hirst & Hirst, 1910, *Amblyomma parvitarsum* Neumann, 1901, *Bothriocroton undatum* Fabricius, 1775 (published as *Aponomma decorosum*), *Dermacentor andersoni* Stiles, 1908, *Hyalomma aegyptium* Linnaeus, 1758, *Hyalomma dromedarii* Koch, 1844, *Hyalomma marginatum* Koch, 1844, *Rhipicephalus bursa* Canestrini & Fanzago, 1878, *Rhipicephalus evertsi* Neumann, 1897, *Ixodes percavatus* Neumann, 1906, (sensu lato), *Ixodes woodi* Bishopp, 1911 and *Otobius megnini* (Dugès, 1883) (Dantas-Torres et al., 2009; Amorim et al., 2013; Serra-Freire et al., 2015; Faccini-Martínez et al., 2021; Labruna et al., 2020).

On the other hand, only three exotic tick species have become established in Brazil and are currently considered pests and vectors of pathogens for domestic animals: *Dermacentor nitens* Neumann, 1897, *Rhipicephalus microplus* Canestrini, 1888, and *Rhipicephalus sanguineus* Latreille, 1806 (sensu lato) (Aragão, 1936; Barros-Battesti et al., 2006). It is worth noting that these three exotic species that have become established in Brazil have already been found on humans in different regions of the national territory (Guglielmone & Robbins, 2018).

Although the tick *D. nitens* is a vector of *Babesia caballi* in horses and has been sporadically recorded on humans in Brazil by Guglielmone et al. (2006) and Serra-Freire (2010, 2014), according to Guglielmone & Robbins (2018), human parasitism by this tick species in Brazil requires confirmation, a fact recently confirmed by Szabó et al. (2020). On the other hand, the species *R. microplus*, which has already been found on humans working in direct contact with livestock, is important for animal health because it transmits *Anaplasma marginale*, *Babesia bovis* and *Babesia bigemina* (Labruna et al., 2005; Barros-Battesti et al., 2006). Lastly, the tick *R. sanguineus* s. l. not only transmits *Anaplasma platys*, *Babesia canis vogeli*, *Babesia gibsoni*, *Ehrlichia canis* and *Hepatozoon canis* to dogs, but has also been found to be infected with the bacterium *R. rickettsii*, thus representing a public health threat in this country (Barros-Battesti et al., 2006; Ogrzewalska et al., 2012).

Based on the above-mentioned facts, the identification of *D. variabilis* on a Brazilian traveler is an indication not only of the imminent risk of the introduction and establishment of exotic ticks in Brazil but also of the possibility of importation of pathogens from other countries, since this tick species is native to North America and is an important vector for *R. rickettsii* on that continent.
